# Roles of CD4+ T cells as mediators of antitumor immunity

**DOI:** 10.3389/fimmu.2022.972021

**Published:** 2022-09-09

**Authors:** Dmitriy S. Kravtsov, Amy K. Erbe, Paul M. Sondel, Alexander L. Rakhmilevich

**Affiliations:** ^1^ Department of Human Oncology, University of Wisconsin, Madison, WI, United States; ^2^ Department of Pediatrics, University of Wisconsin, Madison, WI, United States

**Keywords:** CD4+ T cells, adoptive cell therapy, MHC, cytotoxicity, immunotherapy

## Abstract

It has been well established that CD8+ T cells serve as effector cells of the adaptive immune response against tumors, whereas CD4+ T cells either help or suppress the generation of CD8+ cytotoxic T cells. However, in several experimental models as well as in cancer patients, it has been shown that CD4+ T cells can also mediate antitumor immunity either directly by killing tumor cells or indirectly by activating innate immune cells or by reducing tumor angiogenesis. In this review, we discuss the growing evidence of this underappreciated role of CD4+ T cells as mediators of antitumor immunity.

## Introduction

Engaging adaptive immunity for the control, regression, and complete elimination of tumors has been a primary focus of both preclinical and clinical immunotherapeutic approaches for treating a variety of cancers. It is well established that CD8+ cytotoxic T lymphocyte (CTL) responses are effective at tumor clearance in various models, particularly for tumors that express MHC class I (MHC-I) molecules recognized by CD8+ T cells. Another major player in the adaptive arm of the immune system is CD4+ T lymphocytes, a heterogeneous group of T cells with distinct subsets. It has long been established that CD4+ T cells help in the generation of an effective CD8+ CTL response (1–4). Additionally, a lot of attention has been devoted to the role and function of CD4+ regulatory T cells (Tregs) in cancer (5–8). CD4+ Tregs have a role in cancer progression by suppressing both CD4+ and CD8+ effector responses and exerting various other immunosuppressive effects on the tumor microenvironment (TME). Aside from the helper and suppressive roles of CD4+ T cells in the induction and maintenance of CD8+ CTL responses, evidence continues to accumulate pointing towards a more direct role for CD4+ T cells in antitumor immunity, sparking increased interest in the utility of CD4+ T cells in cancer immunotherapy. However, the mechanisms by which CD4+ T cells mediate tumor clearance instead of, or in addition to, CD8+ T cells are not clearly defined. Determining such mechanisms is of importance for cancer treatment as they will provide insight into when engaging CD4+ T cells, either alone or alongside CD8+ T cells, will mediate effective antitumor immunity. In this brief review, we will describe the evidence in the literature and the mechanisms by which CD4+ T cells primarily mediate antitumor responses and immune memory.

## Roles of CD4+ T cell effectors in humans

A direct role for CD4+ T cells mediating tumor control in the setting of metastatic disease in human cancers has been seen in individual and small cohorts of patients. In one case, adoptive cell transfer (ACT) of *ex-vivo* expanded autologous NY-ESO-1-specific CD4+ T cell clones resulted in long-term complete remission in a patient with refractory metastatic melanoma (9). In another case of a patient with metastatic cholangiocarcinoma, CD4+ T cells in the tumor-infiltrating lymphocyte (TIL) population exhibited cytotoxic potential *in vitro* (10). Adoptive transfer of these cells resulted in tumor regression and disease maintenance in the patient (10). Furthermore, the transferred cells remained in metastatic lesions and immunohistochemical analysis revealed that all tumor lesions were MHC class II (MHC-II) negative, suggesting that these CD4+ T cells were not responding directly to the MHC-II deficient tumor cells (10). Although these findings involve individual patients, they demonstrate examples of the therapeutic potential of CD4+ T cells in controlling advanced disease.

Another study of 4 patients with advanced melanoma found that ipilimumab generated or augmented multiple T helper type 1 (Th1) dominant CD4+ T cell responses, seen by analysis of *in vitro* cultured CD4+ T cells isolated from patients’ peripheral blood (11). Additionally, these CD4+ T cells expressed cytotoxic markers and were able to recognize and kill antigen-presenting cells (APCs) that processed their target peptide (11). These CD4+ T cells were also cytotoxic against an autologous antigen-expressing and MHC-II-positive melanoma cell line in one patient, and blockade of MHC-II on the target cells inhibited this cytotoxicity (11). In another patient, the authors observed that granzyme B, perforin, and the transcription factor Eomes were upregulated in CD4+ T cells after ipilimumab treatment, while PD-1 expression was reduced, suggesting that ipilimumab induces Eomes expression in CD4+ T cells, which in turn may be the driver of increased expression of lytic granules (11). A study with a larger cohort of 18 multiple myeloma patients found naturally occurring cytotoxic CD4+ T cells that expressed granzymes A and B and perforin, were able to kill autologous plasma cells, and were correlated with milder disease, suggesting that CD4+ T cell-mediated cytotoxicity helped control disease (12). These CD4+ T cells also exhibited lower levels of the immune checkpoint molecules PD-1 and CTLA-4 compared to CD8+ CTLs (12).

In 7 patients with localized muscle-invasive bladder transitional cell carcinoma who either did or did not receive anti-PD-L1 immunotherapy before surgical resection, Oh et al. found two cytotoxic CD4+ T cell populations that were increased in bladder tumors while CD8+ T cell populations were similar between tumor and adjacent non-malignant tissues (13). These cytotoxic CD4+ T cells could lyse autologous tumors in an MHC-II-dependent manner (13). The authors also found that cytotoxic CD4+ T cells were clonally expanded, thereby suggesting that they might be recognizing cognate tumor antigens *in vivo* (13). Accordingly, clinical response to anti-PD-L1 treatment was significantly correlated with a cytotoxic CD4+ T cell gene presence in the inflamed phenotype patients as defined by the authors (13). Recently, a new approach of using CD4+ T cells as antitumor effectors in a form of CD4+ chimeric antigen receptor (CAR) T cells has been investigated with promising results showing that they were more effective than CD8+ CAR T cells in eliminating CD19+ target cells in NSG mice (14). Upon comparing different CD4+ T cell phenotypes, CD26^high^ CAR T cells were identified to be more effective at eliminating tumors than Th1, T helper type 2 (Th2) or T helper type 17 (Th17) CAR T cells (15). In another study, T helper type 9 (Th9) CAR T cells were more effective than Th1 CAR T cells (16).

Another study of 32 advanced melanoma biopsies identified a cytotoxic subset within the CD4+ T cell population in different patients and described its genetic signature (17). Peripheral blood tumor-specific CD4+ T cells obtained from *ex vivo* sorted cells from patient samples effectively lysed MHC-II-expressing target cells (17, 18). Interestingly, in comparison to CD8+ T cells, CD4+ T cells showed delayed killing with a higher rate of specific lysis (17). At least in this *ex vivo* model, the cytotoxicity kinetics suggest that CD4+ T cells may contribute to tumor cell killing over a longer period and potentially with higher specificity compared to conventional CD8+ CTLs. CD4+ T cells had a direct, contact-dependent, perforin and granzyme B-mediated cytotoxicity against tumor cells, which was in part dependent on SLAMF7 (17). This study confirms previously published reports on the role of perforin and granzyme B in CD4+ T cell-mediated cytotoxicity, where inhibitors of granzyme and perforin reduced cytotoxicity of CD4+ T cells (19). Interestingly, data from The Cancer Genome Atlas RNA sequencing dataset showed a significant relationship between SLAMF7 positivity and better prognosis in melanoma and other cancers (17). Finally, the authors also discovered cytotoxic CD4+ T cell subsets using databases for breast, head and neck, and hepatocellular cancer, further supporting the existence of cytotoxic CD4+ T cell subsets among TILs in human cancers (17). Oh et al. noted that the cytotoxic subset had higher maximal production of interferon-gamma (IFNγ) and tumor necrosis factor alpha (TNFα) in this study and postulated that in this setting, other pathways may contribute to tonic target cell killing by cytotoxic CD4+ T cells in addition to contact-dependent granule exocytosis (18). To summarize, the presence of human CD4+ T cells capable of killing cancer cells *via* perforin and granzyme B pathways has been established by several independent studies.

## Roles of CD4+ T cells in tumor regression in mice

### CD4+ T cells in ACT experiments

Multiple lines of evidence demonstrate the effector mechanisms for CD4+ T cell-mediated rejection of tumors in mice. One such line of evidence can be gathered from ACT experiments. *In vitro*-generated Th17 cells were able to eradicate B16 melanoma in mice, and this effect was reported to be dependent on IFNγ (20). In another study, adoptive transfer of naïve tumor-specific CD4+ T cells eliminated B16 tumors, and the adoptively-transferred cells differentiated into a Th1-like phenotype, expressed cytotoxic molecules, and induced MHC-II expression on tumor cells, suggesting that direct cytotoxicity may at least partially mediate their antitumor effects (21). Similarly, ACT of naïve tumor-specific CD4+ T cells along with CTLA-4 blockade led to regression of large B16 melanomas, and the transferred CD4+ T cells developed cytotoxic activity (22). In this same study, B16 melanoma cells increased MHC-II expression *in vivo* in an IFNγ-dependent manner, and the adoptively transferred CD4+ T cells could kill tumor cells *in vitro* and reject challenge tumors *in vivo* in an MHC-II-dependent manner (22). Additionally, OX40 agonist engagement in the setting of chemotherapy-induced lymphopenia with adoptively transferred tumor-specific CD4+ T cells was effective in treating B16 melanomas and chimeric B16:B78H1 melanomas (23). OX40 engagement induced a cytotoxic CD4+ T cell population that expressed markers of terminal differentiation and memory (23).

Unlike the previous studies that demonstrate a direct cytotoxic capacity against MHC-II-expressing tumors as a possible mechanism of CD4+ T cell-mediated tumor clearance by adoptively transferred CD4+ T cells under lymphopenic conditions, other evidence from ACT experiments suggests that CD4+ T cells can also control tumors without relying on direct tumor lysis. Thus, in the model in which tumor-derived antigen could be presented to T-cell receptor-transgenic (TCR-tg) CD4+ T cells by host but not tumor MHC-II molecules, adoptive transfer of pre-activated CD4+ T cells was able to control the growth of tumors in some lymphopenic hosts (24). These findings show that adoptively transferred CD4+ T cells have the capacity to control tumor growth without direct recognition and killing of tumor cells, such as in MHC-II-negative tumors, or requiring help from other adaptive immune cells. Similarly, adoptively transferred CD4+ T cells specific for an MHC-II-restricted, tumor-specific peptide were found to eliminate MHC-II-negative UV-induced fibrosarcoma 6132A-PRO tumors by an indirect mechanism dependent on IFNγ activation of host cells (25). Another ACT study of immune cells from TCR-tg mice found that CD4+ T cells were more efficient at tumor rejection than CD8+ T cells *in vivo* in 6 different tumor models and required MHC-II expression by host tissue, but not tumor, for clearance (26). The authors hypothesized that CD4+ T cells partner with other innate cells, including natural killer (NK) cells and macrophages, to inhibit tumor growth. Another study using TCR-tg mice and ACT of tumor-specific CD4+ T cells demonstrated that mouse lymphoma and melanoma were rejected through an IFNγ-dependent mechanism that involved indirect activation of CD4+ T cells by secreted antigen expressed on host APCs (27). MHC-II expression on tumors was not required for their rejection, but inducible nitric oxide synthase (iNOS) expression by T cell-activated tumor-infiltrating macrophages was required for tumor cell killing (27). These indirect antitumor effects were observed by ACT of Th1 (28) as well as Th2 (29) CD4+ T cells, suggesting in a view of the Th2-skewing hypothesis of tumor escape (30) that CD4+ T cells have antitumor potential at both earlier as well as later stages of tumor growth. Interestingly, as noted above for CAR-T cells, adoptively transferred Th9 cells were more effective in eliminating large tumors than Th1 or Th17 cells (31). Th9 cells mediated direct granzyme B-dependent cytotoxicity against tumor cells, had upregulated Eomes expression, and were hyperproliferative enabling them to have extended persistence *in vivo* (31). In addition to enhanced efficacy, Th9 cells, but not Th1 or Th17 cells, expressing tumor-specific T cell receptors or CAR were able to eradicate advanced tumors that contained antigen-loss variants (32).

More recently, treatment of a HER2+ breast cancer cell line tumor in a humanized mouse model with a trispecific antibody to HER2, CD3, and CD28 and adoptive transfer of CD4+ T cells demonstrated regression of cancer (33). Blockade of TNFα decreased the antitumor effect by CD4+, but not CD8+, T cells in the presence of the trispecific antibody *in vitro* (33). Additionally, *in vitro* killing was observed after 9 hours, arguing against a large contribution of fast-acting cytotoxic mediators (33). Instead, CD4+ T cells caused target cell arrest in the G1/S phase of the cell cycle in the presence of the trispecific antibody, a finding that was not seen with CD8+ T cells, demonstrating another potential mechanism by which CD4+ T cells may exert antitumor effects, although this remains to be seen *in vivo* (33). To summarize, the data from ACT experiments show that CD4+ T cells can mediate direct tumor killing of MHC-II-expressing tumors and indirect killing of MHC-II-negative tumors, possibly by their activation of macrophages in the TME, which can then mediate killing that is not restricted by tumor expression of MHC I or II.

It is important to remain cautious when interpreting results from ACT studies and extending them to immunocompetent tumor-bearing hosts, as the adoptively transferred CD4+ T cells in these studies were observed to mediate their antitumor effects under lymphopenic conditions. Because ACT regimens utilize lymphodepletion, they create a substantially altered immune environment, which may be a reason for their observed direct effector roles. In fact, ACT of CD4+ T cells was ineffective in controlling tumor growth in immunocompetent hosts (24). In the absence of host T and B cells, immune signaling is different and it could be the case that adoptively transferred CD4+ T cells acquire their direct antitumor capabilities because they are the only adaptive effector cell left. With more immune players present in the immunocompetent host, immune signaling and cellular interactions are vastly more complex. Showing a role for effector cells in both ACT experiments and depletion experiments can provide more convincing results. Thus, vaccination with alpha-galactosylceramide-loaded A20 lymphoma cells elicited effective antitumor immunity against tumor challenge, and depletion as well as adoptive transfer studies revealed an exclusive role of conventional CD4+, but not CD8+, T cells in mediating antitumor immunity (34).

### Cytotoxic CD4+ T cells in mice

The presence and differentiation of CD4+ T cells into cytotoxic subsets in the setting of infection and inflammation was reviewed by Takeuchi et al. (35). As was discussed for human studies and ACT studies, CD4+ T cells can be directly cytotoxic for tumor cells. This was also shown during endogenous immune responses in tumor-bearing mice. Thus, a virus-induced FBL3 tumor in mice, which is normally eliminated by CD8+ T cells, was eliminated by tumor-specific CD4+ T cells that were also shown to produce granzyme B, but only in the absence of CD8+ T cells and Tregs (36). It is known that the FBL3 murine leukemia normally does not express MHC-II molecules *in vitro*; however, the authors observed that after being in the host environment, a proportion of the inoculated MHC-II-deficient FBL-3 cells became MHC-II positive, so direct recognition and killing of the tumor is possible in this case (36). To confirm the *in vivo* killing potential of CD4+ T cells after depletion of Tregs and CD8+ T cells, the authors demonstrated MHC-II-restricted killing by CD4+ T cells (36).

In another model, BALB/c mice with BNL hepatocellular carcinoma (HCC) tumors treated with dendritic cells (DC) fused with BNL cells (DC/BNL) and systemically administered interleukin-12 (IL-12) successfully rejected tumor, and CD4+ T cells appear to be the critical effectors, in that CD4+, but not CD8+, T cell depletion entirely reversed the antitumor effect (37). The CD4+ T cells were shown to possess cytotoxic activity *in vitro* that was suppressed by inhibition of perforin but not Fas ligand (37). After treatment, large numbers of CD4+ T cells and MHC-II-positive macrophages infiltrated tumor tissue (37). Because BNL tumor does not express MHC-II, the authors suggested that tumor killing *in vitro* likely occurred through the presentation of tumor antigens to DC/BNL-primed CD4+ CTLs leading to perforin-mediated MHC-independent killing of nearby MHC-II-negative tumor cells (37). As emphasized by the authors, however, it should be noted that the applicability of these findings is limited by the method of DC vaccine preparation. The fusion of DC and BNL cells allows the ingestion of tumor cell components that may not be ingested normally when DCs encounter tumor cells (37). This method likely allows for the presentation of MHC-II-restricted tumor antigens that may not normally be presented to CD4+ T cells, and this could be responsible for eliciting the cytotoxic CD4+ T cells in this treatment paradigm (37). However, there are several reports describing the activation of CD4+ T cells by DCs in the TME (38, 39). The other major limitation of this study is it does not prove the mechanistic hypothesis presented. Indeed, the same group reported that in another experiment with BNL HCC tumors treated with BNL lysate-pulsed DCs and systemic IL-12 administration, the tumor-suppressive effect required the presence of both CD4+ and CD8+ T cells, and CD8+ T cells were essential, suggesting they are potentially the critical mediators of antitumor activity in this treatment regimen (40).

These results are not surprising as data demonstrate that different treatment paradigms and cancer characteristics or environments affect the transcriptome of cytotoxic CD4+ T cells (18). For example, Oh et al. cite evidence for multiple documented cytolytic effector molecules expressed by CD4+ T cells in various cancer models of non-small-cell lung cancer, colorectal cancer, hepatocellular carcinoma, bladder cancer, osteosarcoma, breast cancer, and head and neck cancer, although the individual roles of some have not yet been directly tested (18). Based on the data reviewed by Oh et al. it should be noted that there are inconsistent findings across human cancers with regards to cytotoxic CD4+ T cell immune checkpoint and transcription factor expression in different studies, suggesting that the limited findings from murine studies are not perfectly translatable to human cancers and further transcriptomic data of cytotoxic CD4+ T cells in murine cancer paradigms may be needed.

### CD4+ T cell-mediated stimulation of innate effector cells

In addition to being directly cytotoxic to tumor cells, CD4+ T cells can mediate antitumor effects indirectly. In particular, CD4+ T cells can mediate antitumor immunity through the stimulation of innate immune cells to attack tumors. As previously mentioned, a role for CD4+ T cells and NK cells was identified in an ACT study, but the exact mechanisms of this interaction were not determined (26).

Using irradiated B16 cells transduced with granulocyte-macrophage colony-stimulating factor (GM-CSF) as a vaccine 2 weeks before tumor challenge, Hung et al. analyzed the effector mechanism of B16 tumor rejection and suggested that CD4+ T cells play a role in recruiting and activating eosinophils and macrophages to control tumors (41). The importance of CD4+ T cells in tumor rejection was demonstrated by the fact that none of the vaccinated CD4 knockout mice rejected the challenge tumor, while all vaccinated wild-type mice did, and a significant portion of the CD8 knockout mice was able to reject the challenge tumor (41). CD4+ T cells produced the Th1 cytokine IFNγ and the Th2 cytokine IL-4, both of which were required for maximal tumor immunity (41). The authors’ results show ample eosinophil and macrophage infiltration into the tumors of immunized wild-type mice, which correlated with their ability to reject the tumor (41). Conversely, eosinophil and macrophage tumor infiltration were absent in vaccinated CD4 knockout mice (41). Importantly, IL-5 knockout mice had reduced survival and no eosinophil infiltration into the tumor challenge site, supporting the well-known role of IL-5 as an eosinophil differentiation and chemotactic factor (41). These data suggest that eosinophils can be important mediators of tumor rejection, at least in this model. In addition, the correlation of complete loss of the antitumor effect with the virtually absent iNOS expression in IFNγ knockout mice in this study suggests that macrophage-derived nitric oxide (NO) may act as an important *in vivo* antitumor effector mechanism (41). In another study, vaccination of mice with an irradiated GM-CSF transduced MHC-I-negative tumor vaccine (B78H1-GM-CSF) protected mice against MHC-I-negative tumor challenge, and depletion of either CD4+ T cells or NK cells completely abrogated tumor rejection (42). These results suggest that both NK cells and CD4+ T cells are each required to mediate rejection of these MHC-I-negative tumor challenges; while the CD4 + T helper cells have likely encountered tumor antigen on host MHC-II-positive APCs, the exact roles of the NK cells and the CD4+ T cells in tumor destruction have not been completely resolved.

Another mechanism of tumor rejection that requires the interaction of CD4+ T cells and macrophages has been described in a series of studies by Bogen and colleagues (27, 43–45). Briefly, using TCR-tg mice and ACT experiments in MOPC315 multiple myeloma, B-lymphoma, and B16 tumor models, the authors described a mechanism in which secreted tumor antigen is taken up by tumor-infiltrating macrophages and presented to CD4+ T cells on MHC-II molecules. This in turn stimulates Th1 cells to produce IFNγ, which activates macrophages to an M1 phenotype. These macrophages then secrete NO through the iNOS mechanism, which diffuses to neighboring tumor cells and triggers their apoptosis. This mechanism is independent of MHC-II expression on tumor cells but dependent on tumor-specific antigen secretion for subsequent presentation by host APCs (43). In another study, peritoneal exudate cells with an M2 phenotype could be repolarized to an M1 phenotype and function through interaction with antigen-activated CD4+ Th1 cells *in vitro* (46). Adoptively transferred tumor antigen-specific CD4+ Th1 cells also accumulated in tumors and their presence increased the expression of M1-associated genes and proteins on tumor-associated macrophages *in vivo* (46).

Intratumoral IL-12 treatment of xenografts of non-disrupted pieces of human primary non-small cell lung tumors implanted into SCID mice was shown to suppress tumor growth (47). The treatment promoted survival of human leukocytes within the TME. While both human CD4+ and CD8+ T cells secreted IFNγ, the authors found that CD4+ T cells accounted for the majority of IFNγ production which correlated with the antitumor effect (47). The antitumor effect of local IL-12 treatment depended on CD4+ T cells, IFNγ, and NO (47). A later study by this same group confirmed that CD4+ T cells are activated by IL-12 treatment and help cause tumor destruction (48). In summary, indirect antitumor effects of CD4+ T cells by activating macrophages have been established by several research groups using different tumor models.

### CD4+ T cell effect on tumor vasculature

Another mechanism by which CD4+ T cells can slow tumor growth is by inhibiting tumor angiogenesis. An antiangiogenic effect has been observed to occur through the release of IFNγ by CD4+ Th1 cells, but the exact mechanism of action may be distinct when comparing different tumor models. Qin et al. reported that in the Mc51.9 tumor model, mice immunized with irradiated tumor cells and challenged with tumor 2 weeks later remained tumor-free, but depletion of CD4+ T cells eliminated antitumor immunity (49). Tumor immunity required IFNγ receptor expression on nonhematopoietic cells, likely within tumor stroma, and involved inhibition of tumor-induced angiogenesis (49). A later study by the same group found that rejection of different tumors that are primarily controlled by CD8+ T cells was correlated with inhibition of tumor-induced angiogenesis likely *via* an IFNγ-dependent mechanism, suggesting a shared mechanism for tumor rejection by both CD4+ and CD8+ T cells (50). In other tumor models, CD4+ T cell-derived IFNγ could inhibit tumor angiogenesis by acting directly on tumor cells, which requires tumor responsiveness to IFNγ (51). Another study examining the MHC-II-negative CT26 tumor model found that following treatment with peptide immunization in the absence of Tregs, tumor-specific CD4+ T cells were able to reject the tumor (52). This antitumoral effect was dependent on IFNγ production, which exerted a potent antiangiogenic activity (52).

Although the studies above postulated that the inhibition of angiogenesis likely occurred due to the secretion of angiogenesis inhibitors by either tumor cells or surrounding stroma, none of these studies demonstrated this. IFNγ secreted by CD4+ T cells to my induce the expression of interferon-gamma-induced protein 10 (IP-10) and/or monokine induced by IFNγ (Mig) on tumor cells or stroma, leading to inhibition of angiogenesis (53–55). Another possibility by which CD4+ T cell-derived IFNγ might inhibit tumor growth is by acting directly on endothelial cells. By modifying a tumor to secrete IFNγ, Kammertoens et al. showed that IFNγ acted on endothelial cells and caused regression of tumor blood vessels, resulting in the tumor being held in an ischemic state similar to that of non-hemorrhagic necrosis in ischemia, lending to delayed tumor growth (56). Moreover, CD4+ T cells may control tumor growth through vessel normalization, the process which improves tumor vessel perfusion and oxygenation, enhances the efficacy of immunotherapy, and reduces metastasis (57, 58). Recently, IFNγ-positive Th1 cells were shown to play a role in vessel normalization, and checkpoint blockade-induced activation of CD4+ T cells increased vessel normalization (59).

Despite the proposed antiangiogenic mechanisms mediated by CD4+ T cells, the inhibition of tumor angiogenesis by itself is not likely to lead to complete tumor rejection in humans, especially in large and already well-vascularized tumors. Therefore, the inhibition of tumor angiogenesis and vessel normalization by CD4+ T cells is likely an auxiliary mechanism that may be complemented by other antitumor functions of CD4+ T cells and other immune subsets.

### CD4+ T cells as effectors of immune memory in mice

The role of CD4+ T cells in the generation of immune memory and helping CD8+ T effector cells has long been recognized (4, 60, 61). There are also few reports on the ability of CD4+ T cells to function as effector cells in mice rejecting tumor rechallenge. When using cryo-thermal therapy to treat mice implanted with B16F10 melanoma, a strong systemic and melanoma-specific antitumor immune memory was generated and CD4+ T cell depletion abrogated the antitumor memory response in some mice, while CD8+ T cell depletion demonstrated no inhibition of antitumor memory compared to non-depleted controls (62). Interestingly, the authors found that treatment reduced Tregs in the spleen, lung, and blood 21 days after treatment, and in the late stage of treatment (90 days post-treatment), splenic CD4+ T cells were predominantly differentiated into Th1, CD4+ CTL, and T follicular helper subsets and had elevated Eomes expression (62).

In the MB49 bladder cancer model, mice that rejected their tumors following CpG treatment developed tumor-specific immunity which was abrogated by CD4+ T cell depletion but not CD8+ T cell depletion (63). Similarly, using the same mouse model of bladder cancer treated with IL-12 and chitosan, Smith et al. showed that while CD8+ T cells were required for rejection of primary tumor, depletion of CD4+ T cells, but not CD8+ T cells, in the cured mice before and during tumor rechallenge abrogated the memory response, indicating a role for CD4+ T cells as effector cells in the memory immune response (64). The mechanisms for CD4+ T cell effector functions in immune mice, whether they were direct or indirect, were not addressed in these studies.

## Concluding remarks

**Figure 1:**
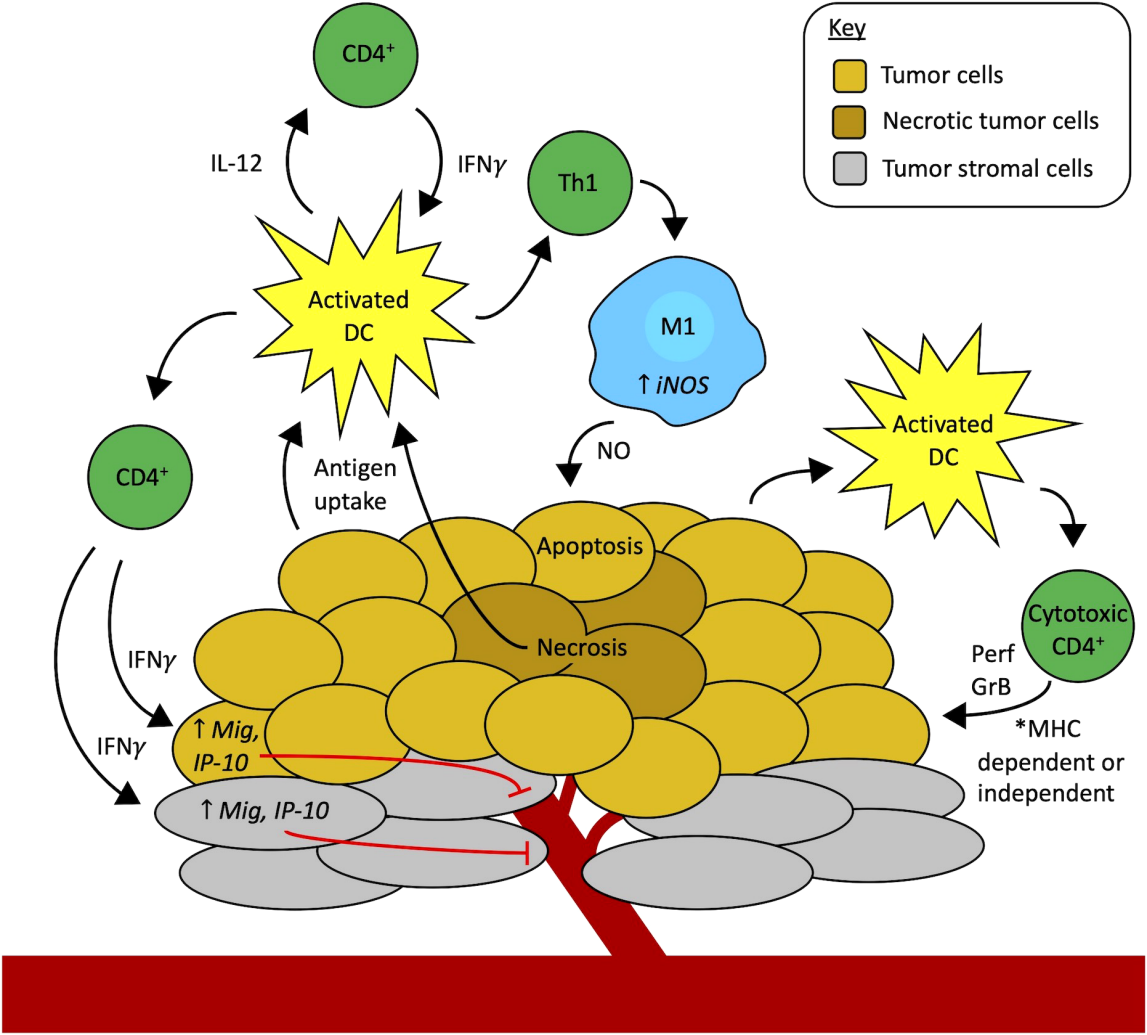
Mechanisms of CD4+ T cells as effectors of antitumor immunity independent of CD8+ T cells. Professional antigen-presenting cells (APCs), like activated dendritic cells (DCs, yellow), in the tumor microenvironment (TME) take up tumor antigens. Starting on the left-hand side of the figure, activated DCs present antigens to CD4+ T cells via MHC-II, in turn activating CD4+ T cells to secrete cytokines like IFNγ, TNFα, and IL-2 that activate effector CD8+ T cells (not shown here). Additionally, IFNγ from activated CD4+ T cells can act on tumor cells (gold) or stromal cells (grey) to increase the expression of monokine induced by IFNγ (Mig) and interferon-gamma-induced protein 10 (IP-10), causing inhibition of tumor angiogenesis and thus contributing to tumor cell death. Another possibility is that CD4+ T cells can contribute to tumor vessel normalization (not shown here). Activated DCs also secrete IL-12, stimulating CD4+ T cells to secrete more IFNγ which can feed back to activate more DCs, setting up a reciprocal interaction that further amplifies the immune cell activation cascade in the TME. Activated CD4+ T cells that differentiate into the Th1 subtype also release IFNγ that activates macrophages (blue) to an M1 phenotype through upregulation of inducible nitric oxide synthase (iNOS). Nitric oxide (NO) secreted by these M1 macrophages in the TME causes apoptosis of tumor cells. Finally, activated APCs, including DCs, may also stimulate the development of cytotoxic CD4+ T cell subsets. These cytotoxic CD4+ T cells may attack the tumor through two mechanisms. They may directly recognize MHC-II-positive tumor cells *via* their T-cell receptor and use contact-mediated delivery to release perforin (Prf) and granzyme B (GrB) to cause tumor cell death. Alternatively, cytotoxic CD4+ T cells may potentially recognize tumor cells in an MHC-independent fashion (like NK cells do) and use other activating receptors to identify activating ligands for those receptors that are selectively expressed by tumor cells to enable contact-mediated release of Prf and GrB into the tumor cells to eliminate MHC-deficient tumor cells through an MHC-independent mechanism. The ongoing tumor cell death from all these synergistic mechanisms may drive further antigen uptake by APCs and the development of a more robust antitumor immune response in the TME.

A role for CD4+ T cell help in generating potent CD8+ effector cells has long been established. However, the antitumor function of CD4+ T cells in the absence of CD8+ T cells has been reported in several studies, both preclinical and clinical, both for the treatment of primary tumors and rejection of tumor rechallenge in immune mice, in certain situations. In some cases, CD4+ T cells can be directly cytotoxic to tumor cells, and in other cases they act indirectly, either by activating innate immune cells or by reducing tumor angiogenesis as depicted in **Figure 1**. It is not yet clear what determines if CD8+ T cells or CD4+ T cells or both will mediate antitumor immunity: whether this depends on Fas/Fas ligand interactions (37, 65, 66), MHC class I/II expression, the tumor type, the type of treatment, or other mechanisms. Additional experimental evaluation, in these separate settings, is warranted to address these important biological questions.

## Author contributions

DK wrote the review. AE, PS, and AR critically revised the review. All authors contributed to the article and approved the submitted version.

## Funding

This work was supported by Midwest Athletes Against Childhood Cancer; Stand Up 2 Cancer; the St. Baldrick’s Foundation; the Crawdaddy Foundation; and the University of Wisconsin Carbone Cancer Center. This research was also supported in part by public health service grants R35-CA197078, P01 CA250972, and 5P30CA014520-40 from the National Cancer Institute; the National Institutes of Health and the Department of Health and Human Services. The content is solely the responsibility of the authors and does not necessarily represent the official views of the National Institutes of Health.

## Conflict of interest

The authors declare that the research was conducted in the absence of any commercial or financial relationships that could be construed as a potential conflict of interest.

## Publisher’s note

All claims expressed in this article are solely those of the authors and do not necessarily represent those of their affiliated organizations, or those of the publisher, the editors and the reviewers. Any product that may be evaluated in this article, or claim that may be made by its manufacturer, is not guaranteed or endorsed by the publisher.
